# Genetic and Histological Alterations Reveal Key Role of Prostaglandin Synthase and Cyclooxygenase 1 and 2 in Traumatic Brain Injury–Induced Neuroinflammation in the Cerebral Cortex of Rats Exposed to Moderate Fluid Percussion Injury

**DOI:** 10.1177/0963689717715169

**Published:** 2017-06-30

**Authors:** Hideki Shojo, Cesario V. Borlongan, Tadashi Mabuchi

**Affiliations:** 1 Department of Legal Medicine, Interdisciplinary Graduate School of Medicine and Engineering, University of Yamanashi, Chuo, Yamanashi, Japan; 2 Department of Neurosurgery and Brain Repair, University of South Florida Morsani College of Medicine, Tampa, FL, USA; 3 Department of Biochemistry, Interdisciplinary Graduate School of Medicine and Engineering, University of Yamanashi, Chuo, Yamanashi, Japan

**Keywords:** head trauma, brain injury model, neuroinflammation, neurodegeneration, cyclooxygenase, prostaglandin synthase

## Abstract

After the initial insult in traumatic brain injury (TBI), secondary neurodegeneration occurs that is intimately associated with neuroinflammation. Prostaglandin (PG) synthases and cyclooxygenase (COX) 1 and 2 may contribute to inflammation in the brain. Temporal and spatial expression features of PG and COX1 and 2 following trauma may guide the development of antineuroinflammation strategies. Here, we examined PG synthase signaling and COX1 and 2 gene expression levels and COX-1- and 2-positive cell types and their temporal localization in TBI-induced brain in an effort to reveal their participation in the disease’s evolving neuroinflammation. Using brain samples from the cerebral cortex of rats subjected to TBI model of lateral moderate fluid percussion injury (FPI), we sought to characterize the temporal (subacute TBI) and spatial (lateral cortical lesion) brain alterations accompanying the disease progression. Temporal gene expression changes of PG synthase signaling were compared between sham-operated and TBI-treated rats using microarray pathway analysis. Moreover, we examined COX1 and 2 expression patterns and their intracellular distribution in sham-operated and TBI-treated rats by immunohistochemistry. After FPI, COX1 and 2 gene expression levels, and PGE_2_ synthase increased while PGD_2_ synthase decreased, suggesting that PGE_2_ and PGD_2_ afforded contraindicative effects of inflammation and anti-inflammation, respectively. Immunohistochemical analyses showed that both COX1 and COX2 increased in a time-dependent manner in the brain, specifically in degenerating neurons of the cortex. Interestingly, the expression of COX cell type was cell-specific, in that COX1 was particularly increased in degenerating neurons while COX2 was expressed in macrophages. In view of the dynamic temporal and spatial expression of PG, COX1 and 2 gene expression and localization in the injured brain regulating PG synthase and COX1 and 2 activity will require a careful disease-specific tailoring of treatments to abrogate the neuroinflammation-plagued secondary cell death due to TBI.

## Introduction

Traumatic brain injury (TBI) is characterized by severe neurostructural damage that impairs neurological function. Moreover, compelling evidence shows that after the primary insult, secondary neurodegeneration with inflammation occurs, resulting in further progressive brain dysfunction.^1–3^ Additionally, neuroinflammation is recognized as a major contributor to various acute and chronic neurological and neurodegenerative diseases.^4,5^ Cyclooxygenase (COX) is the key enzyme in conversion of arachidonic acid to prostaglandins (PGs), which are both lipid metabolites involved in inflammation.^6^ Two distinct COX isoforms have been characterized, COX1 and 2, which differ in terms of regulatory mechanism, tissue distribution, and preferential coupling to upstream and downstream central nervous system enzymes.^7–9^ In most tissues, COX1 is constitutively expressed and has traditionally been considered to be primarily responsible for homeostatic PG synthesis.^10^ In contrast, COX2 participates in inflammation in various tissues and organs, and is mainly induced in response to inflammatory stimuli.^5^

In the brain, both COX1 and 2 are constitutively expressed. Under physiological conditions, COX1 is mainly expressed in microglia and perivascular cells (a macrophage-derived vascular cell type).^11–13^ Furthermore, recent studies have indicated a proinflammatory role for COX1 in the pathophysiology of acute and chronic neurological disorders.^14–16^ Conversely, COX2 is predominantly expressed in hippocampal and cortical glutamatergic neurons, where it has a pivotal role in synaptic activity, long-term synaptic plasticity,^17^ and neurovascular coupling during functional hyperemia.^18^ Thus, COX2 is linked to anti-inflammatory and neuroprotective properties in several experimental models.^19–21^ However, the roles of COX1 and 2 in neuroinflammation and/or neuroprotection after TBI remain unclear.^5,22–25^ Discrepant roles of COX1 and 2 may be due to differences in type (i.e., innate immune response or ischemia) and cellular target (i.e., neuron or glia) of the insult, which accompany various TBI-induced secondary injuries and temporal sequelae of pathological events. Accordingly, the temporal and spatial cellular expression and distribution of COX1 and 2 after trauma are still unclear.

In our previous study using an experimental TBI model, we identified a causal relationship between apoptosis and inflammatory signaling pathways in the lateral cortex of rat brain following TBI.^3^ Using microarray and histopathological analyses, we showed that TBI-induced apoptosis mediated inflammation at an early stage after trauma. Gene expression profiles revealed various signaling pathways that included not only inflammation and apoptosis but also PG synthases. To date, TBI-induced signaling pathways for PG synthases, which participate in neuroinflammation and/or antineuroinflammation, have not yet been elucidated. Here, we examined PG synthase signaling expression levels and COX1- and 2-positive cell types and intracellular localization in the cerebral cortex of rats subjected to the lateral moderate fluid percussion injury (FPI) model. The expression patterns and cellular distribution of COX1 and 2 following trauma may be important with respect to their potential as therapeutic targets for neurodegeneration that is closely associated with neuroinflammation.

## Materials and Methods

### Subjects

Male Wistar rats were housed in a temperature- and humidity-controlled room on a 12-h light/dark cycle with ad libitum access to food and water. Eight-week-old rats weighing 250 to 300 g were exposed to either sham or TBI surgery. Procedures using animals were approved by the ethics committee, regarding animal experiments at the University of Yamanashi.

### FPI Model of TBI

A fluid percussion device (Model HPD-1700; Dragonfly R&D, Ridgeley, WV) was used to induce TBI in rats.^2,3,26^ Briefly, the device generated a fluid pressure pulse in a 17.5-mm-bore stainless steel cylinder with a 76.2-mm piston stroke. The device was connected to cranial Leur adapters using a flexible high-pressure tube with an inner diameter of 2.3 mm, and the entire system was filled with sterile water. Injury was induced by striking the piston with a weighted metal pendulum released from a predetermined height. This resulted in rapid injection of a small volume of saline into the closed cranial cavity, causing a pulse of increased intracranial pressure that is associated with brain deformation. Pressure pulses were extracranially measured with a pressure transducer, recorded on a digital real-time oscilloscope (TDS210; Sony Tektronix Corp., Tokyo, Japan), and analyzed using WaveStar Version 3.0 software (Sony Tektronix Corp., Tokyo, Japan). Based on prior instrument calibration, pressure pulses were expressed in atmospheres (atm). The fluid percussion device delivered transient pressure fluid pulses of constant waveform and duration (17 to 21 ms) to cause brain injury.

Rats were anesthetized with ketamine (75 mg/kg) and medetomidine (0.5 mg/kg). Animals were placed in a stereotaxic frame, and the scalp and temporal muscle reflected. A craniectomy (diameter 4.8 mm) was performed over the right parietal cortex (3.8 mm posterior to bregma and 2.5 mm lateral to the midline) carefully in order not to penetrate the dura.^27^ A cranial Leur adapter (inner diameter 2.5 mm) was placed on the craniectomy site and tightly mounted to the skull using dental acrylic resin. The animals were housed for 48 h following surgery, and then reanesthetized with ketamine (75 mg/kg) and medetomidine (0.5 mg/kg). The cranial Leur adapter was filled with saline and attached to the fluid percussion device. Animals were subjected to sham (control, *n* = 48) or moderate (3.3 ± 0.3 atm, *n* = 48) fluid percussions. All animals subjected to moderate fluid percussion survived during the experimental period.

### Microarray Analysis

Microarray analysis was performed to characterize gene expression profiles after TBI as described in our previous study.^3^ Briefly, rats were transcardially perfused with physiological saline under general anesthesia at 3, 6, and 12 h after moderate fluid percussion or sham operation. Brains were quickly removed and cut coronally into 2-mm thick sections using a rat brain slicer (Neuroscience Inc., Tokyo, Japan). Injured cortices as seen on stained sections were removed,^2^ placed in RNA*later*^™^ (Takara, Shiga, Japan) overnight at 4 °C, and stored at −20 °C until use.

Polyadenylated RNA was isolated using the QuickPrep micro mRNA purification kit (Amersham Bioscience Corp., Piscataway, NJ) according to the manufacturer’s protocol. Isolated mRNA was quantified by spectrophotometry (BioPhotometer; Eppendorf, Hamburg, Germany), and the purity was measured by the A_260_/A_280_ ratio (1.9 to 2.3).

Microarray analysis was performed using GeneChip Rat Genome 230 2.0 (Affymetrix, Santa Clara, CA) at Kurabo Industries (Osaka, Japan), with microarray raw data placed in the Gene Expression Omnibus (GEO) repository (GEO Accession No.: GSE24047). Data analysis was performed using GeneSpring GX 11.0 (Agilent, Santa Clara, CA) at DNA Chip Research Inc. (Yokohama, Japan) and normalized using a robust multiarray average algorithm. No significant differences were observed in gene expression levels among 3 sham chips (3, 6, and 12 h) in boxplot, cluster, and principal component analyses for quality control. The 3 sham chips were then pooled into one group. Ratios of gene expression levels were compared between chips corresponding to the 3, 6, and 12 h TBI groups and sham group.

In this study, the regulatory pathway of COX1 and 2 was examined based on eicosanoid synthesis and PG synthase regulation in pathway analysis. Pathway analysis of gene expression changes was performed at 3, 6, and 12 h after TBI using GenMAPP 2.1 (MAPPFinder 2.0). Pathway analysis statistical results were considered significant if *Z*-score ≥ 0 and permute *P* ≤ 0.01.

### Histological Procedures

Rats were perfused with 10% neutral buffered formalin at 3, 6, 12, and 48 h after sham or moderate fluid percussion (*n* = 5 per group, total = 40). Brains were immersed in 10% neutral buffered formalin. Sections were dehydrated through a graded alcohol series, cleared with xylene, embedded in paraffin, and cut in 6-µm sections.

Immunohistochemical analysis for COX1 and 2 was performed on antigen-retrieved sections (autoclaved for 10 min in 0.1 M EDTA buffer, pH 8.0) using anti-COX1 (1:100; item no.: 16110; Cayman Chemical, Ann Arbor, MI) and anti-COX2 (1:100; item no.: 1160106, Cayman) antibodies. To determine which cell types express COX1 and COX2, double-staining experiments were performed. In each experiment, anti-COX1 or anti-COX2 antibodies were combined with anti-neuronal specific nuclear protein (NeuN) (rabbit polyclonal, 1:100, Abcam, Cambridge, MA; or mouse monoclonal, 1:100, Millipore, Billerica, MA), anti-ionized calcium binding adaptor molecule 1 (Iba1) (goat polyclonal, 1:100, Abcam), or anti-glial fibrillary acidic protein (GFAP) (rabbit polyclonal, 1:100, or mouse monoclonal, 1:100, Cell Signaling, Beverly, MA) antibodies. NeuN and GFAP are markers for neurons and astrocytes, respectively. Iba1 labels macrophages and microglia. Primary antibodies were detected using appropriate secondary IgG antibodies labeled with Alexa 488 or 594 (1:1,000; Molecular Probes, Eugene, OR). Control experiments were performed with omission of primary antibodies, and yielded immunonegative results. Nuclei were counterstained with Hoechst 3342 (Dojindo, Kumamoto, Japan). To assess the specificity of mouse monoclonal anti-COX1 and rabbit polyclonal anti-COX2 antibodies, western blot analysis was performed using rat TBI brain. COX1 and 2 were clearly detected as single bands by the respective antibodies in the brain tissue (see Supplemental Fig. S1).

Immunohistochemistry for cluster of differentiation 68 (CD68), a macrophage and activated microglia marker, was performed on antigen-retrieved sections (trypsin for 20 min at 37°C) using mouse monoclonal anti-CD68 (1:100; Serotec, Oxford, UK) antibody and a streptavidin–biotin labeling method.^28^ Immunoreactivity was visualized using 3,3′-diaminobenzidine (Dako, Tokyo, Japan). Control experiments were performed with omission of primary antibodies and yielded negative immunostaining. Nuclei were counterstained with hematoxylin.

Histological findings in brain sections were compared between the ipsilateral cortices of operated hemispheres from TBI- and sham-treated animals. Results from rats subjected to TBI with different survival periods were also compared.^3^ Nine areas of the ipsilateral cortex were photographed, and the immunopositive cells were manually counted at 200× magnification (9 areas per lateral cortex in TBI or sham rats, total = 45 areas per group; see Supplemental Fig. S2).

High-power magnification was observed at 600× magnification using the DeltaVision Elite Imaging System (Applied Precision, Issaquah, WA) with a silicon-immersion objective lens (OLYMPUS, Tokyo, Japan). Noise reduction was performed on image data by deconvolution using SoftWoRX Version 5 (Applied Precision). Images were shown as 2-D images using volume viewer.

### Statistical Analysis

Data are represented as mean ± Standard Deviation *(SD)*. For the immunohistochemistry experiments, statistical analysis was performed using analysis of variance (ANOVA) followed by post hoc Bonferroni pairwise tests. Statistical significance was set at *P* < 0.05 for all analyses.

## Results

### TBI Induces Neuroinflammation via PG Synthase Signaling

**Figure 1.**
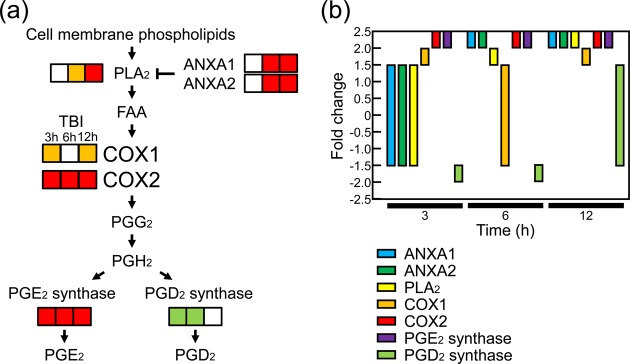
Pathway analysis of traumatic brain injury (TBI)-induced prostaglandin synthase and regulation. Panel (a) shows ratios of gene expression levels were compared between chips corresponding to 3, 6, and 12 h TBI and sham groups. Right-, center-, and left-side columns represent altered gene expression at 3, 6, and 12 h after TBI, respectively. Red, orange, white, and green columns represent fold change (FC) ≥ 2.0, 2.0 > FC ≥ 1.5, −1.5 ≥ FC > 1.5, and −2.0 ≤ FC < −1.5, respectively. The statistical analysis method is described in the experimental procedures. Results were considered significant if *Z*-score ≥ 0 and permute *P* ≤ 0.01. Panel (b) shows the FC of the 7 genes over time.

Here, we analyzed the gene expression profile in the ipsilateral cortices of operated hemispheres at different time points after the TBI, focusing on PG synthase signaling. Pathway analysis of altered transcripts after trauma showed activation of eicosanoid synthesis and PG synthase regulation (Fig. 1) when compared with gene expression levels in cortical sections from control rats (corresponding to 3 sham group chips). Gene expression levels of phospholipase A2 (PLA_2_) increased in a time-dependent manner after the TBI, while *annexin* (ANXA) 1 and 2, and PLA_2_ inhibitors increased after 6 and 12 h (fold change (FC) ≥ 2). Gene expression levels of COX2 and PGE_2_ synthase, which participate in inflammation, significantly increased after 3, 6, and 12 h (FC ≥ 2). Additionally, gene expression levels of COX1 increased at 3 and 12 h (2.0 ≥ FC ≥ 1.5) post-TBI. In contrast, gene expression levels of PGD_2_ synthase, which has an anti-inflammatory role, significantly decreased at 3 and 6 h (FC ≤ −2) after the TBI.

Temporal pathway analysis of altered transcripts after trauma found that *Z*-scores for eicosanoid synthesis were greater than 0 at 3 (FC ≥ 1.5), 6 (FC ≥ 2), and 12 h (FC ≥ 2). Additionally, permute *P* values significantly increased at 3, 6, and 12 h, demonstrating significantly increased eicosanoid synthesis after trauma (Table 1). Furthermore, *Z*-scores for PG synthase regulation were greater than 0 at 3 (FC ≤ −2) and 12 h (FC ≥ 2), with significantly increased permute *P* values at 3 and 12 h, demonstrating that PG synthase regulation was significantly downregulated at 3 h and upregulated at 12 h after trauma. These results indicate that TBI induced neuroinflammation via PG synthase signaling pathways in the ipsilateral cortex.

**Table 1.** Pathway Analysis of Altered Eicosanoid and PG Synthase–Related Gene Expression after TBI.

		*Z*-Score			Permute *P* Value		
MAPP Name	Criteria	3 h	6 h	12 h	3 h	6 h	12 h
Eicosanoid synthesis	FC ≥ 2		3.293	4.030		0.009	0.006
	FC ≥ 1.5	4.724			0.001		
Prostaglandin synthesis regulation	FC ≥ 2			3.375			0.008
	FC ≤ −2	4.964			0.005		

*Note*: Pathway analysis was performed by GenMAPP 2.1 using GeneSpring GX 11.0 (Agilent). Statistical results of pathway analysis were considered significant if *Z*-scores ≥ 0 and permute *P* values ≤ 0.01.

### COX1 and COX2 Expression Increases after TBI with COX1 Primarily Found in Neurons but Rarely in Glial Cells

**Figure 2.**
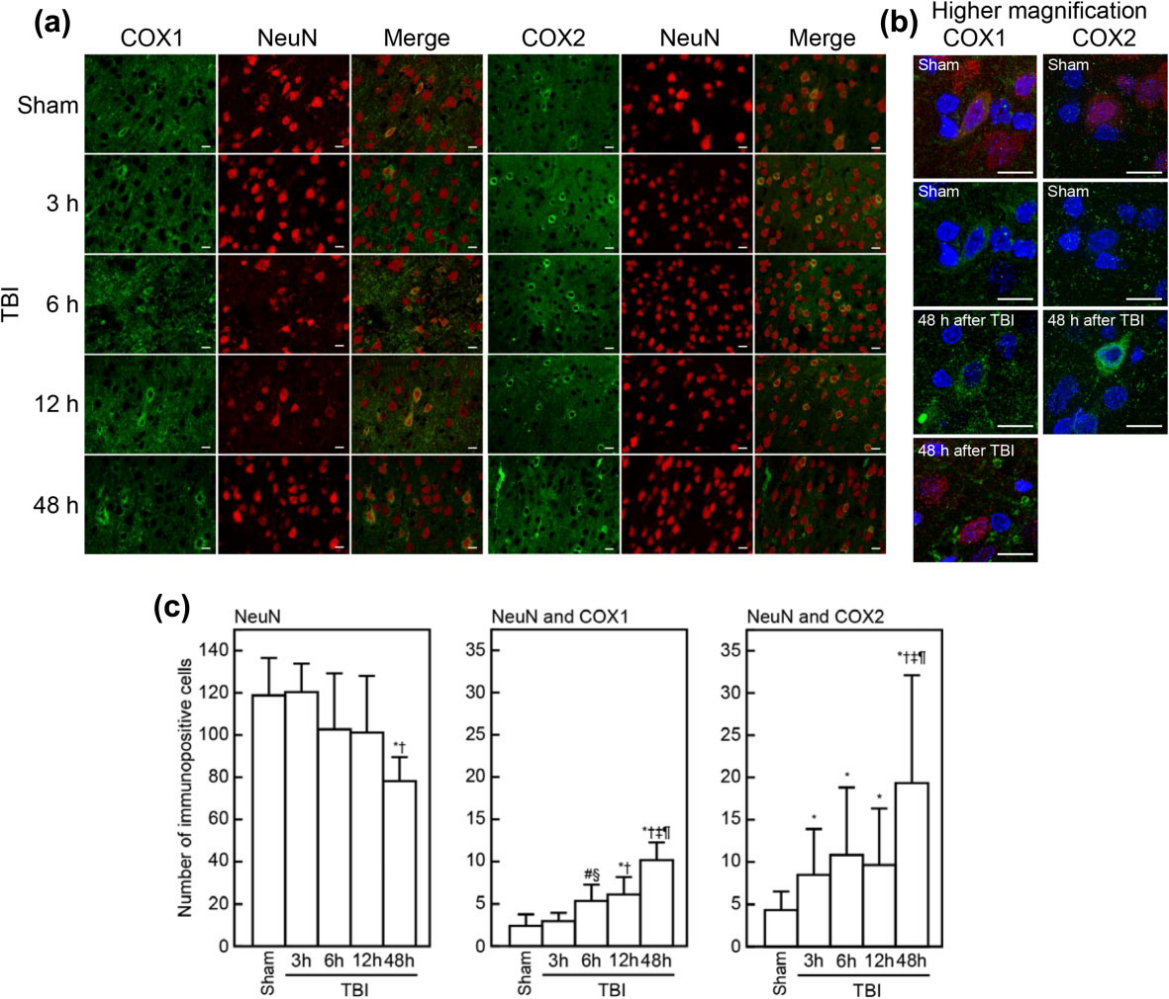
Double-immunostaining of cyclooxygenase (COX) and NeuN in the ipsilateral cortex following traumatic brain injury (TBI). (a) Sham (noninjured control) and histopathological results at 3, 6, 12, and 48 h after TBI. (b) Higher magnification of COX1- and COX2-immunopositive neurons. COX1- and 2-immunopositive cells are shown in green, NeuN-immunopositive cells in red, and nuclei in blue. (c) Number of COX1- and 2- and NeuN-immunopositive cells in the ipsilateral cortex in TBI and sham groups. Mean values ± (*SD, standard deviation*) are shown. Analysis of variance revealed significant main, time, and interaction effects, *F*(3, 45) = 76.08, 41.94, 30.56, *P*s < 0.01). *P < 0.01 compared with the sham group, ^§^*P* < 0.05 compared with the sham group, ^†^*P* < 0.01 compared with the 3 h TBI group, ^‡^*P* < 0.01 compared with the 6 h TBI group, ^∫^*P* < 0.05 compared with the 6 h TBI group, ^¶^*P* < 0.01 compared with the 12 h TBI group. Bars = 15 µm.

The number of COX1-immunopositive cells in the ipsilateral cortex significantly increased at 6, 12, and 48 h after TBI (Fig. 2a). Time-dependent increase in the number of COX1-immunopositive cells after trauma is shown in Fig. 2c. Similarly, COX2-immunopositive cells significantly increased in the ipsilateral cortex at 3, 6, 12, and 48 h after TBI (Fig. 2a). The number of COX2-immunopositive cells increased in a time-dependent manner after trauma (Fig. 2c). NeuN staining was used to identify neurons in double-labeling experiments. NeuN-positive neuronal cell bodies in brain sections of control rats are shown in Fig. 2a. Shrunken neurons were identified in ipsilateral brain sections from injured rats at 3, 6, 12, and 48 h after TBI. In addition, the number of neurons decreased in a time-dependent manner in the cortical area immediately adjacent to the core of the TBI injury in comparison with sham animals.

COX1 was observed in neurons in the brain of control and TBI rats as revealed by the colocalization of COX1 and NeuN in double-staining experiments (Fig. 2a). The number of COX1-immunopositive neurons in the ipsilateral cortex significantly increased at 6, 12, and 48 h after TBI in a time-dependent manner (Fig. 2c).

Higher magnification analysis showed the COX1 immunoreactivity as a granular pattern on cell bodies and their processes. COX1 immunoreactivy significantly increased at 6, 12, and 48 h after TBI, and this increase was time-dependent (Fig. 2b). Additionally, COX1-immunopositive dot-like structures were seen around neuronal cell bodies and dendrites at 48 h after trauma. These results indicate a significant increase in COX1 in degenerating neurons in the cerebral cortex following TBI.

**Figure 3.**
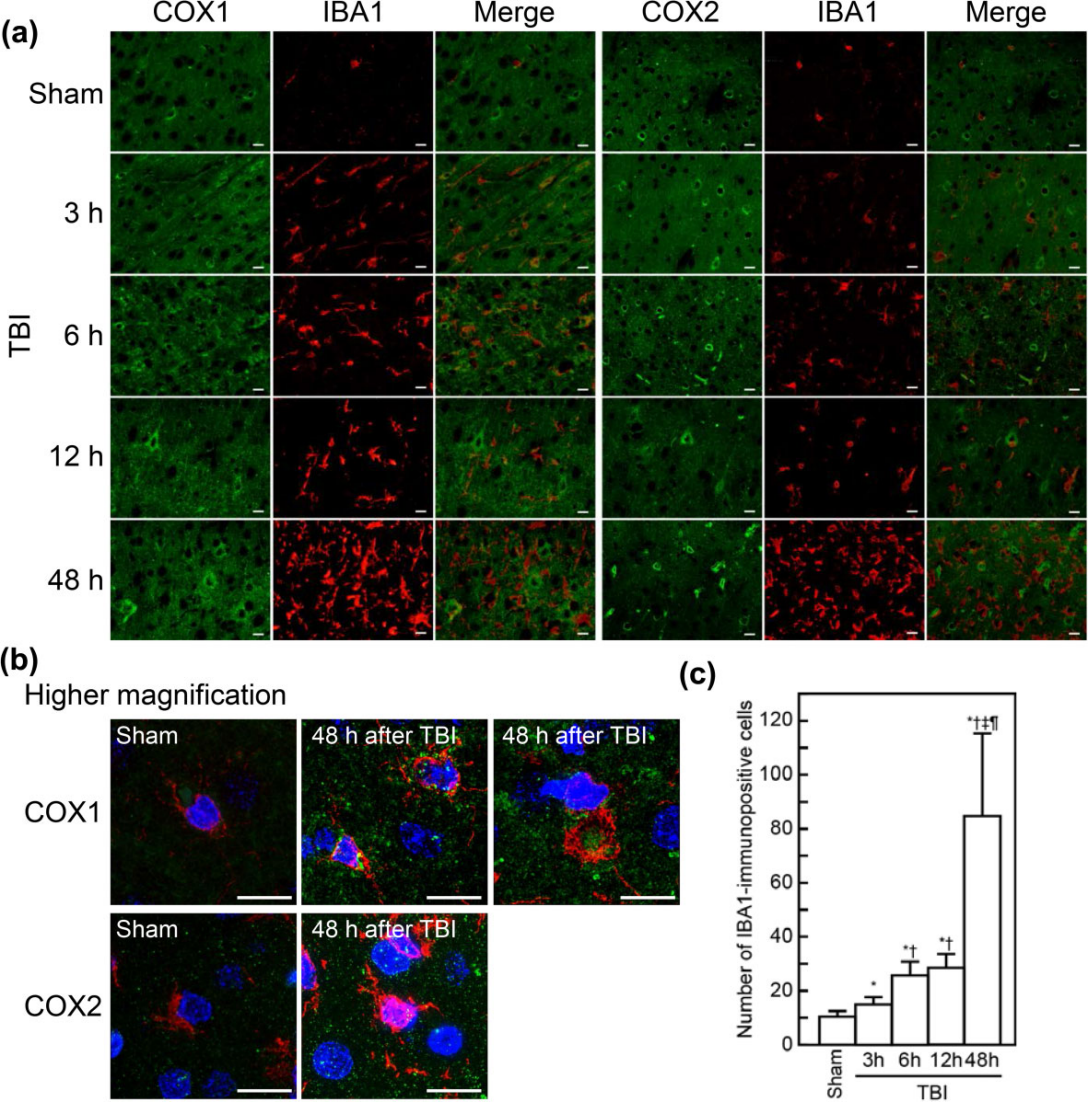
Double-immunostaining of cyclooxygenase (COX) and Iba1 in the ipsilateral cortex following traumatic brain injury (TBI). (a) Sham (noninjured control) and histopathological results at 3, 6, 12, and 48 h after TBI. (b) Higher magnification of Iba1-immunopositive cells. COX1- and COX2-immunopositive cells are shown in green, IBA1-immunopositive cells in red, and nuclei in blue. (c) Number of Iba1-immunopositive cells in the ipsilateral cortex in TBI and sham groups. Mean values ± (*SD, standard deviation*) are shown. Analysis of variance revealed significant main, time, and interaction effects, *F*(3, 45) = 59.13, 32.35, 29.17, *P*s < 0.05). *P < 0.01 compared with the sham group, ^†^*P* < 0.01 compared with the 3 h TBI group, ^‡^*P* < 0.01 compared with the 6 h TBI group, ^¶^*P* < 0.01 compared with the 12 h TBI group. Bars = 15 µm.

**Figure 4.**
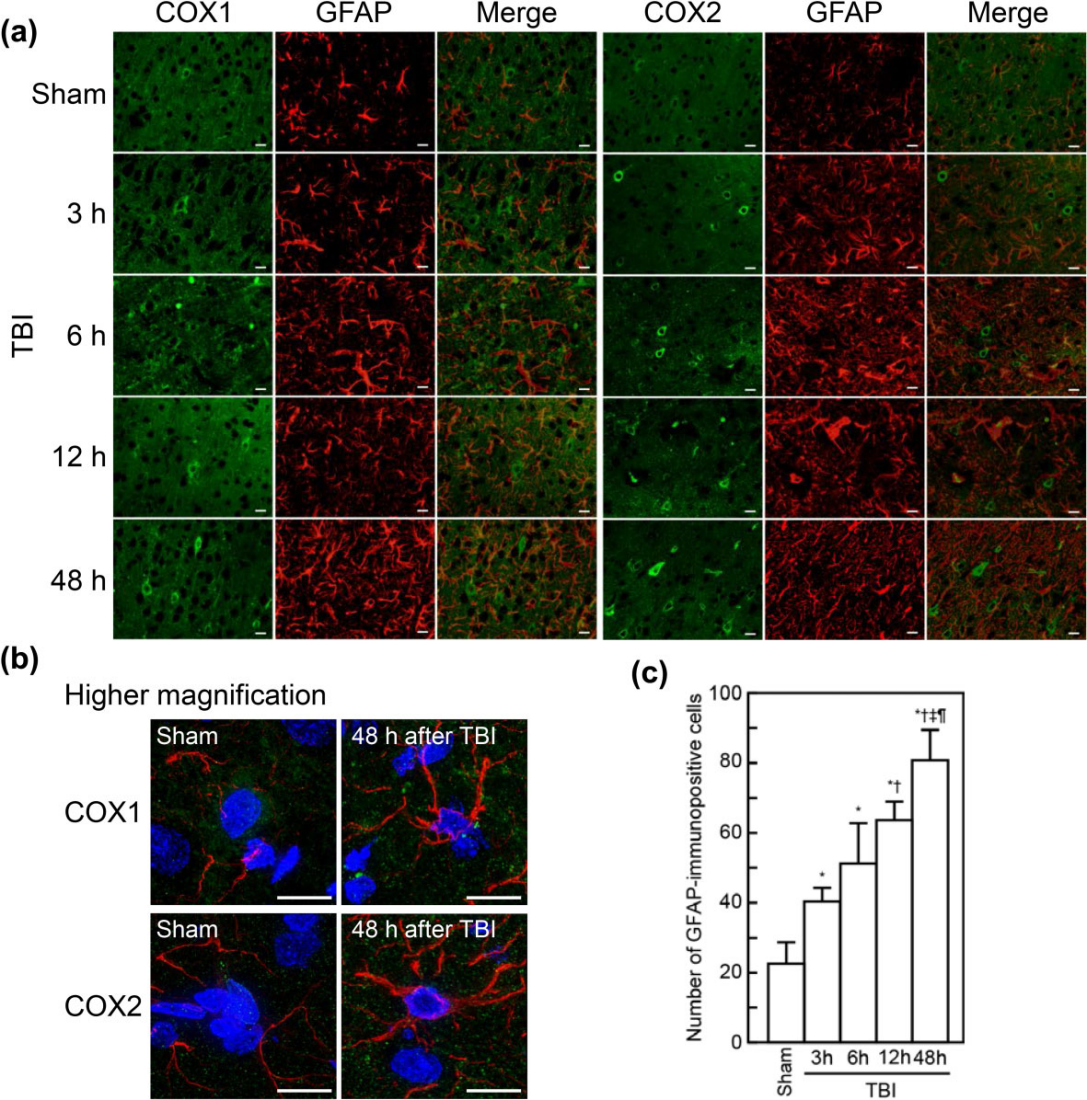
Double-immunostaining of cyclooxygenase (COX) and GFAP in the ipsilateral cortex following traumatic brain injury (TBI). (a) Sham (noninjured control) and histopathological results at 3, 6, 12, and 48 h after TBI. (b) Higher magnification of GFAP-immunopositive cells. COX1- and COX2-immunopositive cells are shown in green, GFAP-immunopositive cells in red, and nuclei in blue. (c) Number of GFAP-immunopositive cells in the ipsilateral cortex in TBI and sham groups. Mean values ± (*SD, standard deviation*) are shown. Analysis of variance revealed significant main, time, and interaction effects, *F*(3, 45) = 65.38, 37.44, 30.62, *P*s < 0.05). *P < 0.01 compared with the sham group, ^†^*P* < 0.01 compared with the 3 h TBI group, ^‡^*P* < 0.01 compared with the 6 h TBI group, ^¶^*P* < 0.01 compared with the 12 h TBI group. Bars = 15 µm.

Iba1 antibody labels microglia and/or macrophages. Iba1-positive cells were hypertrophied and significantly increased in the lateral cortex of the injured hemisphere at 3, 6, 12, and 48 h after the trauma (Fig. 3a). The number of Iba1-immunoreactive cells increased with time after injury (Fig. 3c). Iba1-immunopositive cells were observed in the ipsilateral cortex after TBI including COX1-immunoreactive regions (Fig. 3b). However, higher magnification analysis of Iba1 and COX1 double-staining found no colocalized cells in control or injured rat brains. Similarly, GFAP-immunoreactive astrocytes in the ipsilateral cortex were hypertrophied and significantly increased at 3, 6, 12, and 48 h after trauma (Fig. 4a). The number of GFAP-immunoreactive astrocytes also increased with time after injury (Fig. 4c). Moreover, COX1 and GFAP double-staining showed that COX1 was not expressed in astrocytes during our experimental period (Fig. 4b).

### COX2 Increases in Neurons and CD68-Positive Cells after TBI

COX2 and NeuN double-staining showed that COX2 significantly increased in neurons in the ipsilateral cortex at 3, 6, 12, and 48 h after TBI (Fig. 2a). The number of COX2-immunopositive neurons increased in a time-dependent manner after trauma but slightly decreased at 12 h after trauma (Fig. 2c). Higher magnification analysis showed that COX2-immunoreactivity, seen as a granular pattern at neuronal cell bodies and their processes, significantly increased at 3, 6, 12, and 48 h after injury, and this increase was time-dependent (Fig. 2b). COX2 increased intracellularly after trauma.

**Figure 5.**
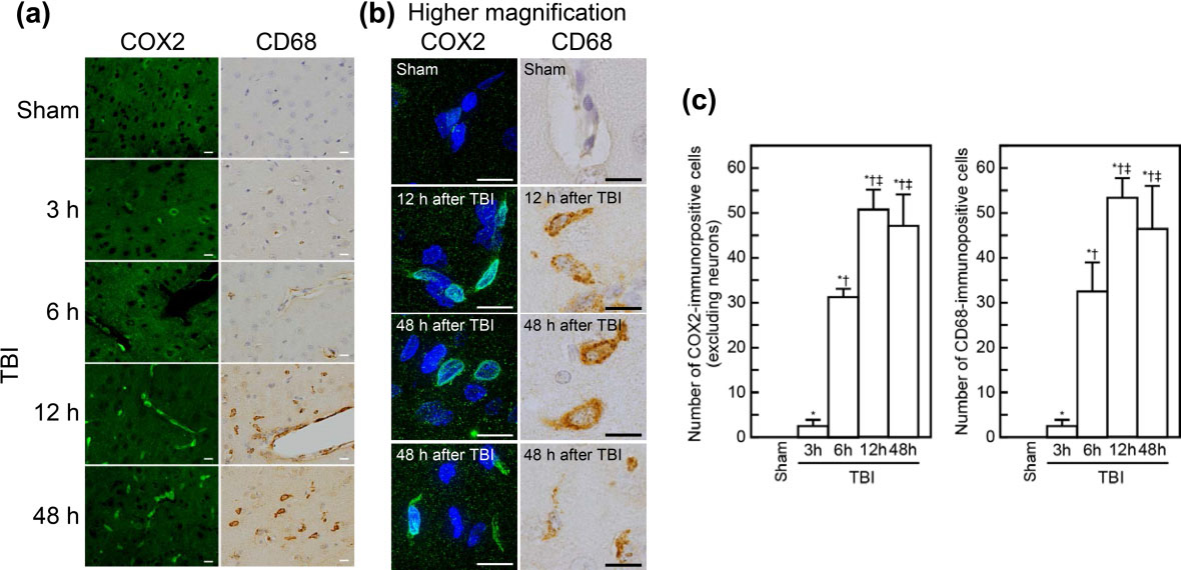
Morphological comparison of cyclooxygenase (COX)2- and CD68-immumopositive cells. (a) Sham (noninjured control) and histopathological results at 3, 6, 12, and 48 h after traumatic brain injury (TBI). (b) Higher magnification of COX2- and CD68-immunopositive cells at 12 and 48 h after TBI. COX1-immunopositive cells are shown in green, CD68-immunopositive cells in brown, and nuclei in blue or dark blue purple. (c) Number of CD68- and COX2-immunopositive cells (excluding neurons) in the ipsilateral cortex in TBI and sham groups. Mean values ± (*SD, standard deviation*) are shown. Analysis of variance revealed significant main, time, and interaction effects, *F*(3, 45) = 47.71, 30.18, 25.12, *P*s < 0.05). *P < 0.01 compared with the sham group, ^†^*P* < 0.01 compared with the 3 h traumatic brain injury (TBI) group, ^‡^*P* < 0.01 compared with the 6 h TBI group (paired Student’s *t* test [two-tailed]). Bars = 15 µm.

Double-staining of COX2 and CD68, which labels microglia and/or macrophages, could not be performed because of differences in antigen retrieval treatments as described in the Method section. However, the spatial distribution of COX2 and CD68-immunopositive cells as well as their morphology was compared. Few CD68-immunopositive cells were found in the cortex of the injured hemisphere at 3 h after TBI (Fig. 5a). CD68-immunopositive cells were observed on the vascular endothelium and around blood vessels at 6 h after injury. The number of CD68-immunopositive cells increased at 12 h and slightly decreased at 48 h after TBI (Fig. 5c).

The morphological features of nonneuronal COX2-immunopositive cells and CD68-immunopositive cells were similar (Fig. 5b). COX2-immunopositive cells resembling CD68-immunopositive cells were observed on the vascular endothelium and around blood vessels at 6 h after injury. Additionally, both the number of nonneuronal COX2-immunopositive cells and CD68-immunopositive cells increased at 12 h and slightly decreased at 48 h after injury (Fig. 5c).

Double-staining experiments with COX2 and Iba1 or GFAP were also performed. No colocalization between COX2 and Iba1 (Fig. 3a and b) or COX2 and GFAP (Fig. 4a and b) was observed in the ipsilateral cortex following TBI, suggesting the COX2 is not present in Iba1-positive cells or astrocytes after TBI.

### COX1 and COX2 Are not Expressed Simultaneously in the Same Cells

**Figure 6.**
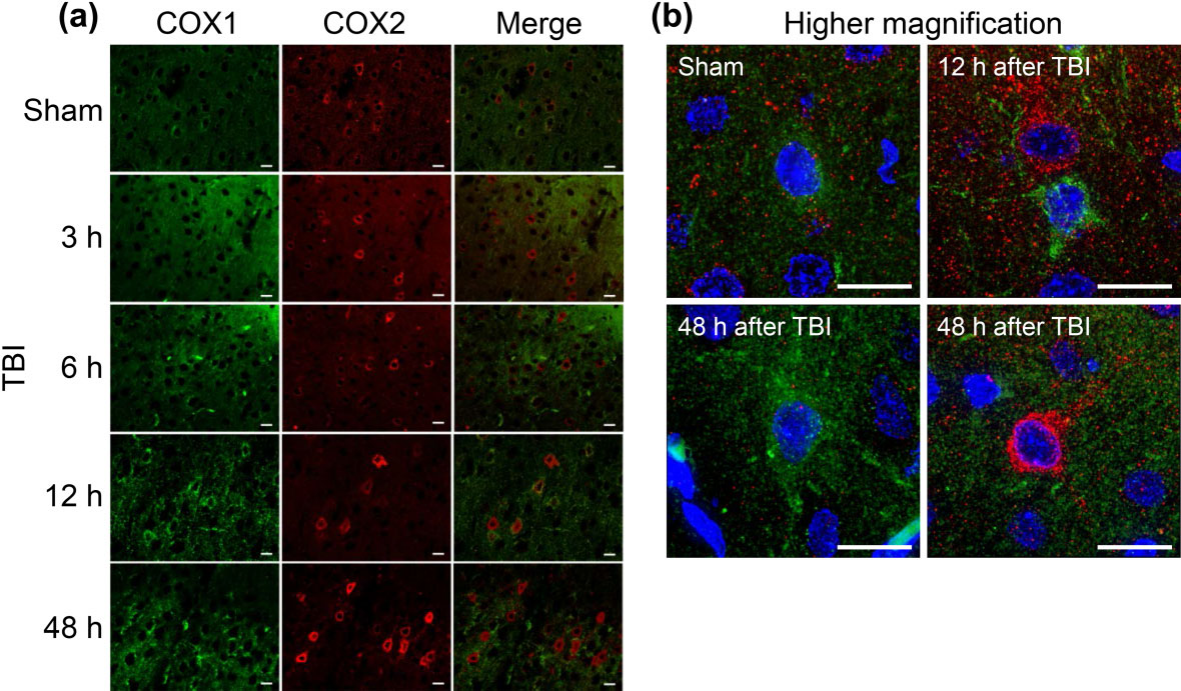
Double-immunostaining of cyclooxygenase (COX) 1 and 2 in the ipsilateral cortex following traumatic brain injury (TBI). (a) Sham (noninjured control) and histopathological results at 3, 6, 12, and 48 h after TBI. (b) Higher magnification of COX1- and COX2-immunopositive cells. COX1-immunopositive cells are shown in green, COX2-immunopositive cells in red, and nuclei in blue. Bars = 15 µm.

An increase in neuronal COX1 and 2 was observed in the ipsilateral cortex after TBI (Fig. 2a and b). To examine whether COX1 and 2 are present in the same cells after the injury, double-staining experiments were performed. The majority of the cells did not colabel with COX1 and COX2, and only sporadic cells were observed to express both COX types, suggesting that many cells do not display simultaneous upregulation of both enzymes following TBI during our experimental period (Fig. 6a and b).

## Discussion

TBI-induced neuroinflammation causes neurodegeneration and neuronal dysfunction. That COX1 and 2 contribute to inflammation and/or anti-inflammation in the brain advances the notion that they may be important therapeutic target for sequestration of neuroinflammation in the disease progression. The present study was designed to reveal the temporal (subacute TBI phase) and spatial (lateral cortical lesion) expression features of PG synthase and COX1 and 2 following trauma. Here, we found that neuroinflammation coincided with alterations in PG synthase during the subacute TBI phase, and was accompanied by increased COX1 and 2 expression in specific cell types with distinct intracellular localization.

The FPI model of TBI produces focal and diffuse brain damage reminiscent of the pathology seen in human TBI. Severity of this TBI model can be manipulated to generate mild, moderate, and severe brain alterations, thereby manifesting a continuum of injury severities. Nonetheless, no single TBI animal model, including this FPI approach, can fully capture the pathological manifestations of human TBI. Despite this limitation, the use of animal models of TBI can represent a specific aspect of human TBI pathology, allowing investigations into the potential mechanisms of cell death underlying the disease. Depending on location of primary injury site, subsequent hemorrhagic insult, blood brain barrier destruction, and inflammation, which is the subject of the present study, the moderate severity FPI model employed here can produce brain pathological and behavioral deficits that approximate symptoms in human TBI.

In our previous study using an experimental TBI model, we performed microarray gene expression profile analysis.^3^ Time-dependent gene expression profiles revealed involvement of various signaling pathways including not only inflammation and apoptosis but also PG synthase. Here, time-dependent gene expression analyses showed significantly increased COX2 and PGE_2_ synthase gene expression levels at 3, 6, and 12 h after trauma (Fig. 1). Additionally, COX1 gene expression levels increased at 3 and 12 h after trauma. In contrast, PGD_2_ synthase gene expression levels significantly decreased at 3 and 6 h after trauma. Temporal pathway analysis of altered transcripts after trauma revealed significantly activated eicosanoid synthesis after trauma (Table 1). Furthermore, *Z*-score and permute *P* values indicated that PG synthase regulation was significantly downregulated at 3 h and upregulated at 12 h after trauma. Downregulation of PG synthase at 3 h after trauma may correspond with PGD_2_ synthase gene expression. Because PGE_2_ and PGD_2_ participate in inflammation and anti-inflammation, respectively, our time-dependent gene expression profile analyses indicate that neuroinflammation is mediated by PG signaling pathways in the ipsilateral cortex of the injured hemisphere during the subacute phase of TBI.

Schwab et al. reported that the perikarya and processes of large neurons express COX1 in normal and injured brains of human patients.^29^ Neuronal COX1 expression was not restricted to lesional or perilesional areas. Our results also show that COX1 is expressed in neurons in sham and injured rat brain. Moreover, COX1 significantly increased in the ipsilateral cortex of the injured hemisphere after trauma, with the number of COX1-expressing neurons significantly increasing in a time-dependent manner. COX1-immunopositive fluorescence exhibited a granular pattern at neuronal cell bodies and dendrites after trauma, and was present as dot-like structures around neuronal cell bodies and dendrites at 48 h after TBI. Our previous study showed that degenerating axons and their terminals display as dot-like structures and granular patterns in the ipsilateral cortex of the injured hemisphere at 2, 15, and 30 days after trauma.^2^ Neuronal cell bodies suffer atrophy and necrosis either directly or indirectly after FPI, therefore synaptic terminals detach from degenerated neuronal cell bodies in the cortex,^30–32^ which may promote neurological deficits in rats.^26^ Taken together, our results indicate that increased COX-immunoreactivity is associated with neurodegeneration in the cerebral cortex following TBI, which may reflect neuronal and synaptic dysfunction. Thus, after trauma, COX1 in neurons induces PG synthase expression and likely participates in progression of chronic neuroinflammation.

In terms of neuroinflammation immunohistochemical assays, Iba1-immunopositive cells significantly increased in a time-dependent manner and were hypertrophied (Fig. 3) in the ipsilateral cortex of the injured hemisphere. In contrast, CD68-immunopositive cells appeared on the vascular endothelium and around blood vessels at 6 h after TBI (Fig. 5). The number of CD68-immunopositive cells increased at 12 h but slightly decreased at 48 h after TBI (Fig. 5). These Iba1- and CD68-immunopositive cells may represent a combination of activated microglia and macrophages.^33^ Indeed, considering the expression patterns of Iba1 and CD68 following FPI from our previous reports,^2,3^ these two types of neuroinflammatory cells are most likely activated microglia and macrophages, respectively, during this early stage of TBI. Similarly, GFAP-immunopositive cells, which represent activated astrocytes, also significantly increased and were hypertrophied in the ipsilateral cortex of the injured hemisphere (Fig. 4). Likewise, the number of GFAP-immunopositive cells increased over time after trauma. Presynaptic terminals withdrew from cell bodies or dendrites of degenerated neurons, and were replaced by astrocyte and microglia processes. This phenomenon is known as synaptic stripping, and depresses synaptic function and impairs neuronal function.^30,34^

Recent studies have shown that under physiological conditions, COX1 is mainly expressed in brain microglia and perivascular cells (a macrophage-derived vascular cell type).^11–13^ Additionally, COX1 is detected at the perinuclear zone and cytoplasm in porcine and human cerebral microvessel endothelial cells.^35^ Schwab et al. reported prolonged accumulation of COX1-expressing macrophages and transient upregulation by endothelium in human and rat with brain injury.^29,36^ The observed COX1 expression in macrophages may signal an induction and exacerbation of neuroinflammation after trauma.^5,37^ Interestingly, we found no COX1 and Iba1 co-expressing cells in brain sections from sham-operated, control rats. In contrast, some Iba1-immunoreactive cells (including those in COX1-immunoreactive regions) were observed in injured rat brain after trauma. COX1-immunoreactive regions likely represented tissue and cell debris from the initial insult as well as erythrocytes due to bleeding, prompting activated microglia and/or macrophages to sequester foreign substances and remove dead cells by phagocytosis.^38,39^ Consequently, our results suggest that microglia and macrophages remove COX1-induced degenerated neurons by phagocytosis negating the proinflammatory response routinely ascribed to COX1 and PG upregulation. Such phagocytotic process leading to the lack of COX1 expression in microglia warrant additional studies.

We also found significantly increased COX2 expression in neurons of the ipsilateral cortex of the injured hemisphere after TBI. The number of COX2-immunopositive neurons increased in a time-dependent manner after trauma, although it slightly decreased at 12 h after trauma. Similar to COX1, COX2-immunopositive fluorescence was observed as a granular pattern at neuronal cell bodies and dendrites. Pilipović et al. reported that TBI induced COX2 overexpression and Fluoro-Jade B-stained neurodegeneration.^40^ Although Günther et al. replicated these findings, increased COX2 expression in male rats did not correlate with Fluoro-Jade B-stained neurodegeneration.^41^ Our results show that COX2-immunopositive fluorescence did not form dot-like structures on degenerated axons or around neuronal cell bodies and dendrites. Surprisingly, we found COX2 distinctly increased intracellularly compared with COX1 expression. These discrepant COX1 and 2 expression patterns suggest divergent regulatory mechanisms mediating specific COX types. The *COX1* promoter lacks a TATA or CAAT box, has a high GC content, and contains several specificity protein 1 (SP1) elements. In contrast, the *COX2* gene has several transcriptional regulatory elements, including nuclear factor-κB, SP1, a TATA box, CAAT enhancer–binding protein β, and cAMP response element–binding consensus sequences, and interacts with transacting factors generated by multiple signaling pathways.^42,43^ Our temporal and spatial immunohistochemical analyses revealed that COX1 and 2 increased in different cell phenotypes after trauma, with varying patterns of elevation during the subacute phase of TBI. These results suggest that COX1 and 2 play distinct roles in neuroinflammation and neurodegeneration after trauma via their respective regulatory mechanisms.

CD68-immunoreactive cells were observed on the vascular endothelium and around blood vessels in the ipsilateral cortex of the injured hemisphere after TBI. We were unable to perform double-labeling experiments with CD68 and COX2 due to differences in the antigen retrieval protocols for these two antibodies. However, nonneuronal COX2-immunopositive cells were morphologically similar to CD68-immunopositive cells, which are most likely infiltrated macrophages as described above. The number of nonneuronal COX2-immunopositive cells and CD68-immunopositive macrophages increased at 12 h and slightly decreased at 48 h after TBI. COX2 was distinctly increased intracellularly but was not observed in Iba1-immunopositive microglia or GFAP-immunopositive astrocytes during our experimental period. In contrast, Strauss et al. reported that COX2 increased in glial cells.^44^ Recent studies using COX2 genetic deletion and/or pharmacological inhibition experiments have shown that COX2 activity is necessary to switch off neuroinflammation,^20,45–49^ suggesting that COX2-derived products mediate a protective effect in the progression and/or resolution of inflammation in the brain after endotoxin activation of innate immunity.^27,28,50–53^ Macrophages and/or microglia activation in the endotoxin challenge neuroinflammation model is well established.^54,55^ Our results suggest that macrophages, but not microglia or astrocytes, prevent neuronal inflammation via COX2 activity in the injured brain after trauma. Macrophages are a major source of inflammatory cytokines such as tumor necrotic factor and interleukin-1α and β. Our previous study found that macrophages participate in neuroinflammation via inflammatory cytokines during the early period after trauma.^3^ Taken together, these results suggest that the expression of COX2 in macrophages during the first 3 to 48 h postinjury likely contributes to proinflammation rather than anti-inflammatory processes.

Our time-dependent gene expression profile analysis indicates that neuroinflammation is caused by PG synthase signaling pathways in the ipsilateral cortex of the injured hemisphere after trauma. Furthermore, COX1 and 2 significantly increase at degenerating neurons, and these immunopositive cells increase in a time-dependent manner during the 3 to 48 h postinjury period. Our approach has limitations in that we are not able to determine the exact roles of COX1 and 2 after TBI using microarray gene expression profiling and immunohistochemical analysis. Nevertheless, our results suggest that, although the COX1 and COX2 are expressed in different cells types and located in distinct intracellular regions, therapies designed at abrogating neuronal COX1 and 2 activity using appropriate inhibitors as antineuroinflammatory agents may prove effective when initiated at the subacute TBI phase.

## Conclusions

Time-dependent gene expression profile analysis suggests that neuroinflammation is caused by PG synthase signaling pathways in the lateral cortex of the impacted hemisphere during the 3 to 48 h postinjury period. COX1 and 2 display specific cellular and intracellular distribution patterns after TBI, but both enzymes were found in degenerating neurons. COX1 and 2 seem to cooperatively participate in neurodegeneration during the early period after trauma and are likely to exacerbate neurodegeneration via PG signaling following TBI.
